# Relationship of perceived environmental characteristics to self-efficacy and leisure time physical activity among Asian immigrants in the U.S.

**DOI:** 10.34172/hpp.2020.55

**Published:** 2020-11-07

**Authors:** Jaehyun Kim, Andrew J. Mowen, Benjamin D. Hickerson, Alan Graefe, Melissa Bopp

**Affiliations:** ^1^Department of Recreation, Therapeutic Recreation, and Tourism, State University of New York, College at Brockport, Brockport, NY; ^2^Department of Recreation, Park, and Tourism Management, The Pennsylvania State University, University Park, PA, USA; ^3^Department of Community and Therapeutic Recreation, University of North Carolina at Greensboro, Greensboro, NC, USA; ^4^Department of Kinesiology, The Pennsylvania State University, University Park, PA, USA

**Keywords:** Environment, Self-efficacy, Physical activity, Immigrants

## Abstract

**Background:** Given the high prevalence of physical inactivity among Asian immigrants and its potential negative effects on health, more attention should be paid to identifying factors that might affect their participation in leisure time physician activity (LTPA). This study examined how perceived environmental characteristics are related to self-efficacy and LTPA among Asian immigrants in the United States.

**Methods:** This cross-sectional study collected data from an on-line survey administered through Qualtrics, a web-based survey software company. In the data analysis, 512 Asian immigrants were included. As independent variables, we assessed perceived environmental characteristics with regard to the perceived accessibility of LTPA-related sites and neighborhood quality. As mediating and outcome variables, we measured self-efficacy and LTPA, respectively. Using AMOS version 22, a path analysis was conducted to measure model fit.

**Results:** The perceived accessibility of the LTPA-related sites (b = 0.10, P = 0.04) and self-efficacy(b = 0.26, P = 0.001) were positively related to LTPA. Perceived neighborhood quality (b = 0.11,P = 0.012 was positively associated with self-efficacy, yet not directly associated with LTPA. Self-efficacy mediated the relationship between the perceived accessibility of LTPA-related sites and LTPA (estimate=0.036, 95% CI=0.015–0.067, P = 0.003).

**Conclusion:** This study suggests that perceiving easy access to LTPA-related sites and living in quality neighborhoods can encourage people to participate in LTPA more often by increasing their self-efficacy toward LTPA. These findings highlight a need for public infrastructural investments to improve accessibility to LTPA-related resources and neighborhood quality, which can potentially increase self-efficacy and promote LTPA among Asian immigrants.

## Introduction

In 2018, 46.7% of adults in the U.S. were not regularly physically active at the recommended level for health.^1^ The prevalence for physical inactivity is even more marked among underserved populations, such as immigrant populations, in the U.S.^2^ Indeed, Asian Americans have consistently reported a lower prevalence of meeting leisure time physical activity (LTPA) guidelines than non-Hispanic Whites.^3-6^ Studies have also found that, in general, more constraints related to LTPA (e.g., feeling unwelcome, distance to leisure resources) have been reported among ethnic minorities, especially among Asian and Hispanic/Latino individuals, than White individuals.^7,8^ Given the high prevalence of physical inactivity among Asian immigrants and its potential negative effects on health, more attention should be paid to identifying factors that might affect their participation in LTPA.

Bandura^9^ indicated that self-efficacy is an important prerequisite for adopting healthy behaviors (e.g., LTPA). For the purpose of this study, self-efficacy is defined as an Asian immigrant’s confidence in adhering to LTPA in the face of difficult barriers. A number of studies have shown that immigrants with high levels of self-efficacy were significantly more likely to be physically active than immigrants with low self-efficacy.^10,11^ Daniel et al^12^ revealed that self-efficacy was more strongly associated with LTPA than educational attainment, general health status, and depression among Asian Indian immigrants in the U.S. As such, it is imperative to examine factors that can enhance self-efficacy levels among Asian immigrants.

Prior research has examined how the physical environment correlates to LTPA across diverse ethnic groups. Specifically, convincing evidence for positive associations have been found between LTPA and the availability of LTPA facilities^13,14^ and proximity to LTPA facilities.^15^ For example, Taylor and his colleagues^16^found that improvements in physical environments for physical activity, such as more bicycle trails, more senior recreation centers, and free gyms, appeared to be important for LTPA. In addition, certain neighborhood characteristics, such as sidewalks, heavy traffic, street lights, unattended dogs, and neighborhood crime rates, have been found to be associated with LTPA.^13,14,17,18^ In previous immigrant studies, perceived neighborhood safety was found to be the most commonly reported contributor to LTPA participation.^17,19-23^

As discussed above, numerous studies have examined the relationships between physical environments and LTPA and between intrapersonal factors and LTPA; however, only a few studies^20,24-26^ have collectively considered the extent to which the physical contexts in which LTPA might occur might influence self-efficacy and LTPA. For example, Motl and colleagues^25^ found an indirect effect of the perceived physical environment (i.e., accessibility to physical activity equipment) on adolescent girls’ physical activity through its effect on an intrapersonal factor (i.e., self-efficacy). Davidson et al^20^ identified neighborhood satisfaction and the presence of sidewalks and parks in neighborhoods as significant determinants of self-efficacy and LTPA. Despite the empirical contributions from previous studies, these investigations mainly studied adolescents and older women.

Taken together, previous findings have indicated that the perceived accessibility of LTPA-related facilities, neighborhood safety, and self-efficacy were associated with LTPA.^27,28^ In this sense, it is expected that the perceived accessibility of LTPA-related sites, neighborhood quality, and self-efficacy will play a significant role in promoting LTPA among Asian immigrants. To date, however, few studies^29^ have considered these individual and environmental factors collectively in a sample of Asian immigrants. To address this research gap, we examined the extent to which physical environments (i.e., perceived accessibility of the LTPA sites, neighborhood quality) influenced an intrapersonal factor (i.e., self-efficacy) and behavior (i.e., L TPA).

## Materials and Methods

### Sample and data collection

This cross-sectional study collected date from an on-line survey administered through Qualtrics, a web-based survey software company. This survey was conducted from January 15 to March 30, 2017. Through Qualtrics, the online survey was administered to a nation-wide convenience sample of adult Chinese, Japanese, and Korean immigrants living in the U.S. Specifically, inclusion criteria for the target population of this study were as follows: (1) self-identified original ethnicity of Chinese, Japanese, or Korean; (2) must have been born in his/her country of origin and immigrated to the U.S.; (3) must live in an urban area with a population of 50 000 or more; and (4) must be an adult (i.e., 18-years-old or older) who has permanently settled in the U.S. The respondents’ demographic characteristics are shown in Table 1.

Qualtrics attempts to ensure the quality of the data via a double opt-in process, asking potential respondents to complete the profile survey and verify his/her email address. Thus, it has been shown that online panels, especially Qualtrics, offer high quality and reliable data.^30^ Qualtrics initially recruited 828 Asian immigrants who were interested in participating in the survey. We excluded the respondents who did not meet the inclusion criteria or answered less than 80% of the questions. Finally, 511 of the 828 surveys (completion rate = 61.7%) were included in the data analysis. A priori power analysis with a G*Power (version 3.0) was used to estimate adequate sample size for testing Goodness-of-fit. With an alpha = 0.05, power = 0.8, and expected effect size = 0.50, the proposed sample size was approximately N = 115 for this study. The present study included a total of 511 surveys in the data analysis, which satisfies the aforementioned criteria.

### Measures

Perceived accessibility of LTPA-related sites was assessed with a combination of two measures: (1) the self-rated *importance* of LTPA-related sites and (2) the *availability* of the LTPA-related sites in the respondent’s neighborhood.^31^ We selected seven types of LTPA sites: walking trail; parks, playgrounds, or sports fields; swimming pool; public recreation centers; school recreation facilities open to the public; beach, lake, river, or creek; and bicycle paths or bike trails.^32^ *Importance* was measured by asking “Please rate the importance of having access to the listed sites or facilities for you to perform leisure time physical activities you like, regardless of whether your neighborhood has them” with each site listed. Each item was assessed with a 5-point Likert-type scale (1 = not at all important to 5 = very important). To assess the *availability* , the respondents were asked to indicate whether they have those sites in their communities (1 = yes to -1 = no). Finally, in order to create a *perceived accessibility* variable, we multiplied the *importance* by the *availability* and then added ‘Five (5)’ to all values so as to have only positive values in the data. Thus, higher scores in the range of -5 to 5 indicated higher accessibility of LTPA sites in the neighborhood. In this study, the *importance* and *availability* scales yielded a Cronbach’s α of 0.85 and 0.76, respectively.

In order to measure neighborhood quality, seven characteristics (i.e., well-maintained sidewalks, enjoyable scenery, less traffic, street lights, dogs on leashes, good air quality, and low neighborhood crime activity) were derived from Brownson et al’s study.^33^ Using this characteristic, *existence* was assessed through the following question: ‘‘Does your neighborhood have...?’’ The respondents were to answer either yes or no. The items were summed and higher scores in the range of 0 to 7 referred to more favorable perceptions of neighborhood quality. The seven items in this scale yielded a Cronbach’s α of .71.

Self-efficacy for LTPA was measured using 13 modified items derived from Barriers Self-Efficacy Scale (BARSE).^34^Examples of the items include “I believe that I could participate in leisure time physical activity if I had to do the activity alone.” and “I believe that I could participate in leisure time physical activity if the weather was very bad.” The respondents were asked to rate their degrees of confidence by specifying a number from (0) “not at all confident” to (100) “highly confident” with (50) “moderately confident” falling in the middle. The items were summed and divided by the total number of items. Thus, higher scores in the range of 0 to 1300 indicated higher levels of self-efficacy among Asian immigrants. The self-efficacy scale yielded a Cronbach’s α of 0.91.

LTPA was assessed using the Global Physical Activity Questionnaire (GPAQ), in which the respondents were asked to report the number of days and duration of the vigorous and moderate intensity leisure activities that they undertook in a typical week. Vigorous and moderate intensity referred to 8.0 and 4.0 METs, respectively. Continuous scores for LTPA were calculated as total MET-minutes per week as in the following:

We considered the respondents’ demographics (i.e., age, gender, annual household income, and length of residence in the U.S.) to be the control variables. In prior research, these specific demographic factors were shown to affect LTPA among immigrant populations.^35-37^

### Data analysis

The data was analyzed using PAWS^®^ Statistics 18 (IBM Corporation Armonk, NY, USA) and AMOS version 22.0 (Amos Development Corporation, Meadville, PA, USA). First, the descriptive statistics for the demographic (Table 1) and study variables (Table 2) was generated. Second, the Cronbach’s alpha coefficients were used to measure the internal consistency of the observed variables. A value of 0.70 or higher is considered as an acceptable reliability coefficient.^38^ A path analysis using AMOS was conducted to test the relationships among perceived accessibility of the LTPA sites, perceived neighborhood quality, and self-efficacy among Asian immigrants. The model fit was assessed using χ^2^statistic, SRMR, RMSEA, CFI, and GFI. For SRMR and RMSEA, values of 0.06 or less indicated good model fit, while for CFI and TLI, values of at least 0.95 indicated good fit.^39^ Following model testing, bootstrapping procedures^40^ was used to examine whether LTPA had a significant indirect effect on the relationship between emotional support and mental health.

## Results

### Study participants

Table 1 describes socio-demographics of the sample of Asian immigrants in this study. The largest household income group was $75 000 or more (58.5%), followed 21.3% who reported annual household income from $50 000 to $74 999. The mean age was 46 years. The most common age group was 36 to 50 years old (42%), followed by 18-35 years old (24%) and 51-64 years old (21%). The most common educational level attained by Asian immigrants was a Bachelor’s degree (43.2%), followed by a master’s degree (25.2%) and a doctorate degree (11.7%). The sample of 511 participants was comprised of 344 females (67.3%), and 167 males (32.7%).

### Correlation analysis

Table 3 shows the bivariate correlations among the variables. Among the Asian immigrants, the perceived accessibility of the LTPA sites (r = 0.13, *P* < 0.001) and self-efficacy (r = 0.27, *P* < 0.001) were positively correlated with LTPA. In this study, perceived neighborhood quality (r = 0.06, *P* > 0.05) was not correlated with LTPA. Moreover, perceived accessibility of the LTPA sites (r = 0.17, *P* < 0.001) and perceived neighborhood quality (r = 0.15, *P* < 0.001) were positively correlated with self-efficacy. As shown above, some correlations coefficients were weak, but significant. The results may be because the sample size is large enough to make a small effect significant.

### Path analysis

The study model provided an acceptable fit to the data (χ^2^(*df* =4) = 4.133, *P* = 0.388; RMSEA = 0.008; SRMR = 0.013; CFI = 0.998; TLI = 0.997). The results from the path analysis (Table 2) indicated that the perceived accessibility of the LTPA sites (*b* = 0.10, *P* < 0.05) and self-efficacy (b = 0.26, *P* < 0.05) had direct effects on LTPA. However, perceived neighborhood quality was not associated with LTPA (*b* = 0.02, *P* > 0.05). The results also indicated that both the perceived accessibility of the LTPA sites (*b* = 0.14, *P* < 0.05) and neighborhood quality (*b* = 0.11, *P* < 0.05) had direct positive effects on self-efficacy.

Using a bootstrapping method,^40^ we conducted a mediation test for the pathway from perceived accessibility of LTPA sites to LTPA through self-efficacy. The results (Table 4) showed a significant indirect effect of self-efficacy on the relationship between the perceived accessibility of the LTPA sites and LTPA (estimate = 0.036, 95% CI = 0.015–0.067, *P* < 0.05). This means that the perceived accessibility of the LTPA increased the levels of LTPA self-efficacy and ultimately, led to LTPA participation among Asian immigrants.

**Figure 1:**
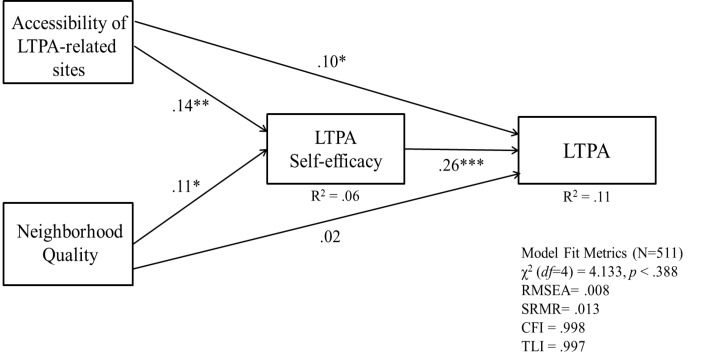
Final path model of leisure time physical activity among Asian immigrants.* *P* < 0.05. **P < 0.01 ***P < 0.001

Overall, the model explained 6% of the variance in self-efficacy and 11% of the variance in LTPA. A final path model with the standardized coefficients and the square root (*R*^2^) is presented in Figure 1.

## Discussion

This study aimed to examine LTPA-related physical and neighborhood environments as potential environmental factors that might affect self-efficacy and LTPA. One of the most unique and interesting findings was that the perceived accessibility of LTPA-related sites (e.g., walking/bike trails, parks/sports fields) was positively related to LTPA, both directly and indirectly, through its positive effect on self-efficacy. That is, Asian immigrants who believed that they had more access to LTPA-related sites were more likely to have self-efficacy and participate in LTPA more often. Moreover, we found that a better quality of neighborhood, such as neighborhoods with less traffic, well-functioning streetlights, and lower criminal rates, encouraged people to participate in LTPA by their improving self-efficacy for LTPA. Our findings were aligned with previous studies that suggested that perceived environment can directly and indirectly affect LTPA behaviors through individual-level perceptions (i.e., self-efficacy).^24-26,41^ Indeed, a recent review study^41^ identified both direct and indirect effects of environmental accessibility (e.g., walkability) on LTPA through increased self-efficacy. The authors also suggested that assessing both self-efficacy and environmental factors has been a common practice when explaining LTPA. In light of these findings, a need exists for developing more comprehensive interventions to address both individual-level perceptions and environmental factors influencing physical activity behaviors.

This study suggests practical implications related to promoting LTPA among Asian immigrants. First, a need exists for public infrastructural investments to improve accessibility to LTPA-related resources and of neighborhood quality. Prior research has suggested that local infrastructure investments to improve access to parks, bicycle paths, sports facilities, sidewalks, and public lighting can promote LTPA among residents and, subsequently, improve their health.^15,18,42-44^ PPrior to infrastructure investments, however, it will be essential for policy-makers and public health professionals to determine specific LTPA-related sites and neighborhood characteristics associated with self-efficacy and LTPA.^45,46^ Such information may inform practitioners and policy-makers about what types and levels of investments should be their priorities.

Considering that our study measured the *perceived* accessibility of LTPA-related facilities and resources (rather than a direct mapping of these resources), it is possible that the respondents were simply not aware of these resources, even though they actually had them in their communities. Individuals are unlikely to use facilities and programs if they are not fully aware of the amenities and programs offered.^47^ It is, therefore, essential to increase the awareness of accessible LTPA-related resources in neighborhoods. Researchers have suggested that media-based campaigns, such as through print materials, local radio and television, and community events, are effective in reaching large numbers of people and, thus, increasing public awareness of accessible LTPA-related sites.^26,44,48^

We found that self-efficacy was most strongly related to LTPA and, as such, improving LTPA self-efficacy among Asian immigrants is another critical implication. With regard to LTPA promotion, prior studies have emphasized the importance of effective interventions to increase individuals’ self-efficacy levels.^18,19,43^ For example, Ashford and colleagues^49^ reviewed 27 interventions to identify specific intervention techniques used for changing participants’ self-efficacy toward physical activity. They revealed that certain interventions, such as vicarious experiences (i.e., seeing others perform physical activities), feedback on physical activity performances from interventionists, and helping identify barriers to physical activity, were found to be effective for increasing physical activity self-efficacy. Consideration of such information could help inform public health professionals about how to develop intervention programs to promote self-efficacy and LTPA among Asian immigrants.

Several limitations exist within the present study. First, the design of the present study was cross-sectional; thus, no causality can be made between the study variables. Instead, the relationship between the variables should be considered reciprocal. For example, this study found that those participants who perceived having a higher level of accessibility to LTPA-related sites were more likely to engage in LTPA. However, it may also be that those individuals who participated in LTPA more often were simply more aware of the LTPA-related sites available to them. Future work, especially prospective studies, could examine the extent to which physical environmental features influence LTPA over time. Second, all of the physical environmental variables assessed in this study were self-reported. It would be interesting if future research could compare objective and perceived accessibility of LTPA-related facilities and examine the relative contribution of those to LTPA. This effort could further clarify the influence of perceived environments on health behaviors among Asian immigrants. Finally, the questionnaires were only written in English; thus, those individuals who were not proficient in English might have been excluded from this study. Given that Asian immigrants often face language barriers,^4,50^ our findings may not be generalizable to all Asian immigrants living in the U.S. Future research could use translated survey instruments to address this issue.

## Conclusion

This study provides evidence that perceiving easy access to LTPA-related sites and living in quality neighborhoods can encourage people to participate in LTPA more often by increasing their self-efficacy toward LTPA. In particular, we confirmed that self-efficacy plays a significant mediating or direct role in promoting LTPA among Asian immigrants. Collectively, the present study expanded the literature by exploring the relationship among perceived physical environments, self-efficacy, and LTPA in a sample of Asian immigrants. More importantly, these findings demonstrate a critical need for public infrastructural investments to improve the accessibility of LTPA-related facilities and resources and neighborhood quality, which can increase self-efficacy and promote LTPA among Asian immigrants.

## Funding

No funding was received for this study.

## Competing interests

There is no conflict of interest or, alternatively, disclosing any conflict of interest that may exist.

## Ethical approval

This research was exempt from further review by Pennsylvania State University’s Internal Review Board (IRB) because it does not meet the criteria for human involving subjects’ research.

## Authors’ contributions

The first author (JK) proposed a research idea and write on the sections of Background and Theoretical Framework. Then, the first author had worked on analyzing the collected data with the second author (AJM) and fifth author (MB). The third author (BDH) and fourth author (AG) commented on the Methods and Results sections. All authors discussed the results and implications and commented on the manuscript at all stages.

**Table 1:** Socio-demographics of the sample of Asian immigrants

**Variable**	**No. (%) or Mean (SD)**
	**Overall (N = 511)**
Income	
$14,999 or less	21 (4.1)
$15,000 to $29,999	27 (5.3)
$30,000 to $49,999	55 (10.8)
$50,000 to $74,999	109 (21.3)
$75,000 or more	299 (58.5)
Age	46 years (SD=13)
18-35	123 (24)
36-50	215 (42)
51-64	107 (21)
65 and older	66 (13)
Education	
Middle school	5 (1)
High school	92 (18)
Bachelor's degree	221 (43.2)
Master's degree	129 (25.2)
Ph.D.	60 (11.7)
Not sure	4 (<1)
Gender	
Male	167 (32.7)
Female	344 (67.3)
Length of residence in the US (y)	18 (SD=13)

**Table 2:** Pearson correlations of independent variables and leisure time physical activity (LTPA)

**Variable**	**1.**	**2.**	**3.**	**4.**
1. Accessibility of LTPA-related sites	1			
2. Neighborhood quality	0.24**	1		
3. Self-efficacy	0.17**	0.15**	1	
4. LTPA	0.13**	0.08	0.27**	1

* P < 0.05. **P < 0.001.

**Table 3:** Direct effects on leisure time physical activity (LTPA)

**Dependent Variable and Path**			**β (SE)**	**t-value**	**R** ^ 2 ^
**LTPA**					**0.11**
Accessibility of LTPA-related sites	→	LTPA	0.10 (0.04)	2.02*	-
Neighborhood quality	→	LTPA	0.02 (4.11)	0 .41	-
Self-efficacy	→	LTPA	0.26 (0.00)	5.98***	-
**Self-efficacy**					**0.06**
Accessibility of LTPA-related sites	→	Self-efficacy	0.14 (7.97)	3.13**	-
Neighborhood quality	→	Self-efficacy	0.11 (4.11)	2.51*	-

**Table 4:** Mediation test: Total, direct, and indirect effects with 95% confidence intervals

**Effect**	**Estimate**	**95 % CI**
Total effect	0.125*	[0.035; 0.217]
Direct effect	0.089*	[0.003; 0.176]
Indirect effect (through self-efficacy)	0.036*	[0.015; 0.067]

* P < 0.05.
